# *HDACs* Gene Family Analysis of Eight Rosaceae Genomes Reveals the Genomic Marker of Cold Stress in *Prunus mume*

**DOI:** 10.3390/ijms23115957

**Published:** 2022-05-25

**Authors:** Juan Meng, Zhenying Wen, Mingyu Li, Tangren Cheng, Qixiang Zhang, Lidan Sun

**Affiliations:** Beijing Key Laboratory of Ornamental Plants Germplasm Innovation and Molecular Breeding, National Engineering Research Center for Floriculture, Beijing Laboratory of Urban and Rural Ecological Environment, School of Landscape Architecture, Beijing Forestry University, Beijing 100083, China; juanmeng@bjfu.edu.cn (J.M.); zywen1220@163.com (Z.W.); lmy15689087371@163.com (M.L.); chengtangren@163.com (T.C.); zqxbjfu@126.com (Q.Z.)

**Keywords:** histone deacetylase *HDACs* family, phylogenetic analysis, *Prunus mume*, cold stress

## Abstract

Histone deacetylases (HDACs) play important roles in plant growth, development, and stress response. However, the pattern of how they are expressed in response to cold stress in the ornamental woody plant *Prunus mume* is poorly understood. Here, we identify 121 *RoHDACs* from eight Rosaceae plants of which 13 *PmHDACs* genes are from *P. mume*. A phylogenetic analysis suggests that the *RoHDACs* family is classified into three subfamilies, *HDA1/RPD3*, *HD2,* and *SIR2*. We identify 11 segmental duplication gene pairs of *RoHDACs* and find, via a sequence alignment, that the *HDACs* gene family, especially the plant-specific *HD2* family, has experienced gene expansion and contraction at a recent genome evolution history. Each of the three *HDACs* subfamilies has its own conserved domains. The expression of *PmHDACs* in mei is found to be tissue-specific or tissue-wide. RNA-seq data and qRT-PCR experiments in cold treatments suggest that almost all *PmHDACs* genes—especially *PmHDA1/6/14*, *PmHDT1*, and *PmSRT1/2*—significantly respond to cold stress. Our analysis provides a fundamental insight into the phylogenetic relationship of the *HDACs* family in Rosaceae plants. Expression profiles of *PmHDACs* in response to cold stress could provide an important clue to improve the cold hardiness of mei.

## 1. Introduction

Epigenetics occurs at the stage of transcriptional regulation, and it affects the pattern of gene expression [1]. Histone acetylation and deacetylation are a dynamic and reversible epigenetics process to regulate the transcription of genes [2]. Histone acetyltransferases (HATs) bind the acetyl of acetyl coenzyme A (COA) to the N-terminal residues of specific amino acids in histones, facilitating the binding of transcription factors to DNA and activating the transcription of genes, whereas histone deacetylases (HDACs or HDAs) remove the acetyl- from the N-terminal residue of specific lysines to repress gene expression [3,4,5,6,7].

Since the *HDACs* gene (*ZmRPD3*) was isolated in maize (*Zea mays*) [8,9], it has been successively discovered in plants, which is classified into three distinct families: reduced potassium dependence3 (RPD3/HDA1), nicotinamide adenine dinucleotide (NAD)-dependent enzyme silent information regulator 2 (SIR2), and the plant-specific Histone Deacetylase 2 (HD2) family [10,11]. The HDA1 type deacetylases are further divided into three groups (class I, II, and IV) based on conserved domains and a homology of sequences [12]. In *Arabidopsis thaliana*, 18 HDACs were identified, i.e., 12 in the HDA1/RPD3 family, 4 in the HD2 family, and 2 in the SIR family [13]. The HDACs family has also been detected in rice (*Oryza sativa* japonica) [14], poplar (*Populus trichocarpa*) [15], and other plants [16,17,18,19,20,21,22,23]. However, it is unclear how frequently they occur in ornamental plants. 

Previse studies suggested that the *HDACs* family plays an important role in plant growth and development [24,25,26,27]. In a cold vernalization pathway, HDA1/9 interact with VP1/ABI3-LIKE 1 (VAL1/2) and CURLY LEAF (CLF)-polycomb group repressive complex2 (PRC2) to decrease the expression of Flowering Locus C (FLC) [28,29]. Plant response to stress includes multiple processes, involving translational regulation, post-translational regulation, and epigenetic modifications [30,31]. Epigenetic regulation via *HDACs* plays an integral role in plant responses to high or low temperature, salinity, and drought abiotic stresses [32]. In *Arabidopsis*, AtHDAs, AtSin3, AtERF4, and AtERF7 protein complexes inhibit ABA and abiotic stress response through deacetylation modification [33]. HDA9 interacts with WRKY to participate in abiotic stress regulation [34]. In cold stress, HDA6 plays a key role in cold tolerance by regulating the expression of cold stress responsive genes [35,36,37]. The cold-treated *axe1-5* mutant was highly sensitive to freezing temperature (−18 °C) compared to the wild type in *Arabidopsis* [36]. AtHDT1/3/4, the plant-specific HD2 proteins, can interact with RPD3/HDA1 histone deacetylases such as HDA6, indicating that HD2s functionally associate with RPD3-type HDACs in the same multiprotein complex to regulate stress response genes in plants [38,39,40]. Overexpressed AtHDT4 showed a higher tolerance to low temperature and drought abiotic stresses [41].

Mei (*Prunus mume*) is an ornamental woody plant, naturally distributed in southern China. In recent years, some cold resistant cultivars, such as apricot mei ‘Songchun’ and plum mei ‘Meirenmei’ have been cultivated by interspecies hybridization between mei, apricot, and plum. However, the molecular mechanisms underlying the responses of mei to cold stress remain unclear. Besides, the functions of HDACs in ornamental plants growth, development and stress, especially in woody plants, have been less studied. In this study, we first identify *HDACs* family members from 8 Rosaceae plants and further study their gene sequences, phylogenetic tree, expression profiles, and putative function. Itreveal that *PmHDACs* in mei could play an important role in mediating this species’ response to cold stress. Our research provides a comprehensive insight into the phylogenetic relationship of *HDACs* family in Rosaceae plants and expression profiles of *PmHDACs* response to cold stress in mei.

## 2. Results

### 2.1. Identification of Rosaceae HDACs Gene Families

To identify *HDACs* genes, hidden Markov models (HMMs) and BLASTP methods were used. We initially queried a total of 125 members in Rosaceae (Tables S1 and S2), from which 4 were deleted based on sequence alignment and domain confirmation. Finally, a total of 121 *RoHDACs* genes were identified in 8 species including mei, apricot (*Prunus armeniaca*), and Chinese plum (*Prunus salicina*) in the Rosaceae family (Table S3). It suggests that *HDACs* gene numbers in *Prunus* species are relatively similar, most of which contain 13 members, such as mei, Chinese plum, peach (*Prunus persica*), and Somei-Yoshino (*Prunus yedoensis*). There are 12 and 16 members in apricot and sweet cherry (*Prunus avium*), respectively (Table 1). Compared to *Prunus* species, rose (*Rosa chinensis*) and apple (*Malus domenstica*) have more than 20 *HDAC* members, 20 for rose and 21 for apple (Table 1). Previous studies showed that there are 16 members in *P. trichocarpa* [15], all of which, except for *PtHDA912*, are identified in this study. For all 13 mei HDACs, 7 PmHDACs proteins (PmHDA1/6/8-1/8-2/15, PmHDT1/3) are located into nucleus, of which PmHDT1 and PmHDT3 have typical nuclear localization sites (NLS) belonging to HD2 family(-KKAK-) (Tables S3 and S4). Protein molecular weights of PmHDACs range from 26,188.27 to 188,010.2 kDa (Table S4). The characteristic of all putative *HDACs* sequences, predicted conserved domains and other structures of the proteins were listed in Table S3.

### 2.2. Multiple Sequences Alignment, Phylogenetic and Classification Analysis of RoHDACs

**Figure 1:**
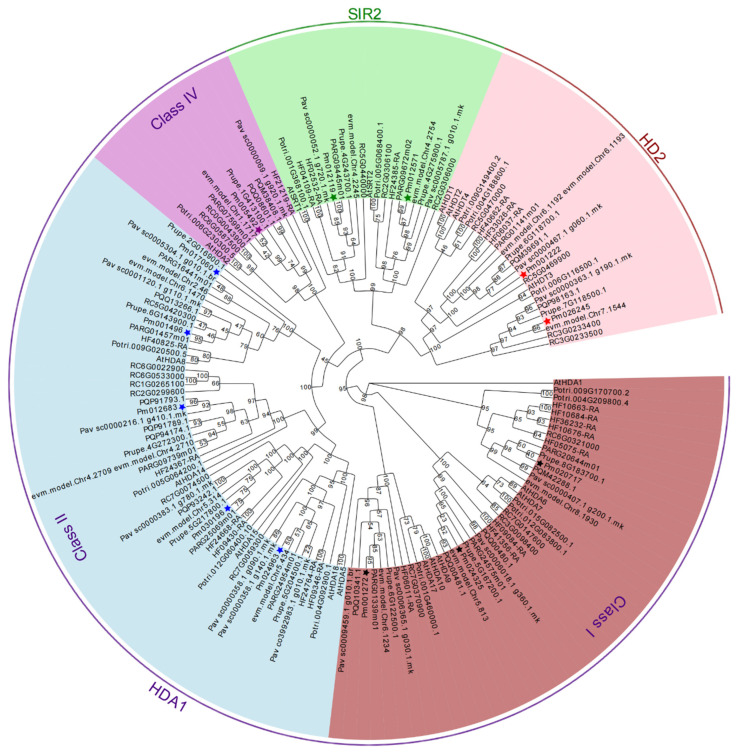
Maximum likelihood phylogenetic tree of histone deacetylases *HDACs* family in 10 plants. The phylogenetic tree in *A.* *thaliana* (At), *P. trichocarpa* (Potri), and Rosaceae plants including *P. mume* (Pm, 13), *P. salicina* (evm), *P. armeniaca* (PAR), *P. persica* (Pruep), *P. avium* (Pav), *P. yedoensis* (PQM), *M. domenstica* (HF) and *R. chinensis* (RC) was reconstructed using IQtree 2.1.3. Bootstrap support of each node was inferred from 1000 replicates. Members marked with asterisks represent mei *PmHDACs*. The red, bule and purple clusters are classified into *HDA1* superfamily named as Class I, II and IV, respectively.

**Figure 2:**
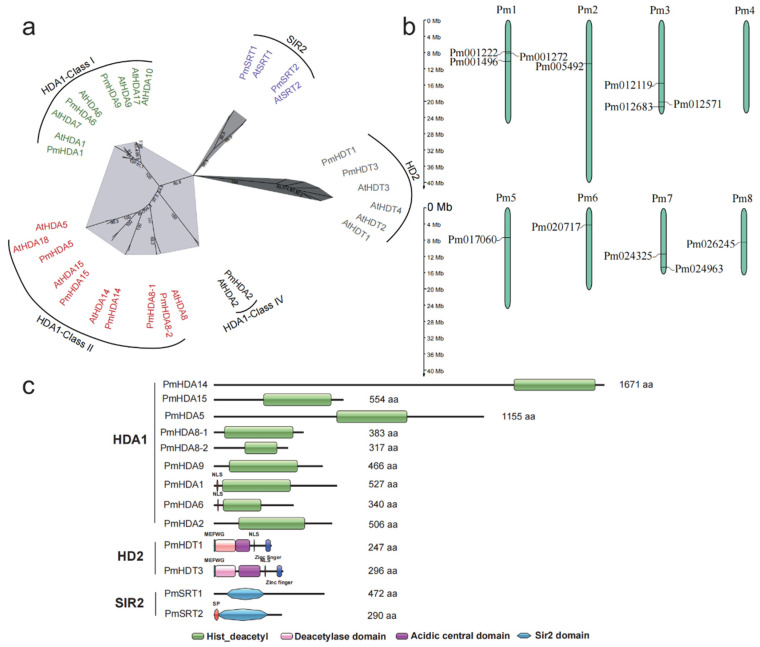
Characteristic analysis of PmHDACs. (**a**) Phylogenetic tree of *HDACs* in *A. thaliana* (At) and *P. mume* (Pm). The unrooted maximum likelihood tree was constructed by IQtree 2.1.3 using complete protein sequences with 1000 bootstraps. (**b**) Gene locations of *PmHDACs* on the mei chromosome; 12 out of 13 PmHDACs are localized on the 8 chromosomes, and 1 is on the scaffolds. The Pm1 to Pm8 represent eight mei chromosomes. (**c**) Conserved domains identified in PmHDACs proteins of three families.

To evaluate the phylogenetic relationship of all putative 121 *RoHDACs*, a maximum likelihood phylogenetic tree of 10 plants was constructed and shown in Figure 1. It indicates that all *RoHDACs* can be classified into three families: *RPD3/HDA1* superfamily, *HD2* family, and *SIR2* family, among which *HDA1* family can further divided into Class I, Class II, and Class IV groups characterized by conserved domain and sequence similarity. We name these 121 *RoHDACs* genes according to the homology with *Arabidopsis HDACs* genes (Table S3). As depicted in Table 1 and Figure 1. The Class I group was composed of three clades based on the core *Arabidopsis* members *AtHDA1/6/9*. There are three *Arabidopsis* orthologues genes (*HDA1/6/9*) in mei, apricot, plum, and peach; four genes in sweet cherry, Somei-Yoshino and rose; and eight in apple. Among them, *PavHDA9* contained two homologous genes, namely *PavHDA9-1/9-2*, *PyHDA7* was found to be orthologues of *Arabidopsis AtHDA7,* and *RcHDA6* has two homologous genes, namely *RcHDA6-1/6-2*.There are at most eight *HDA1* genes in apple: three (*MdHDA1-1 to 1-3*) identified to be homologous of *MdHDA1* and two of *MdHDA6*. For the Class II group, five members were identified in five *Prunus* plants including mei, seven members in sweet cherry and rosa, and six members in apple. We found that *HDA8* in most plants consisted of two homologous genes, i.e., namely *HDA8-1/8-2* (Figure 2a), with *HDA8-2* genes having a shorter sequence and sharing a high sequence similarity (Table S3, Figure S1). Most class IV species contain a single *HDA2* gene, and two homologous genes that only appear in Somei-Yoshino and rose. Several *HDACs* with high-similarity short sequences probably display expansion and contraction during the gene family evolution throng sequence alignment analyzed. For example, there are no *HDA15* and *SIR2* homologous genes, but there are three *HDA14* homologies (*PyHDA14-1/14-2/14-3*) in Somei-Yoshino (Table S3, Figure S2). Besides, three high homologous genes (*MdHDA1-1/1-2/1-3*) are confirmed in apple and four homologous genes (*RcHDA14-1/4-2/4-3/4-4*) in rose (Table S3, Figures S3 and S4).

**Figure 3:**
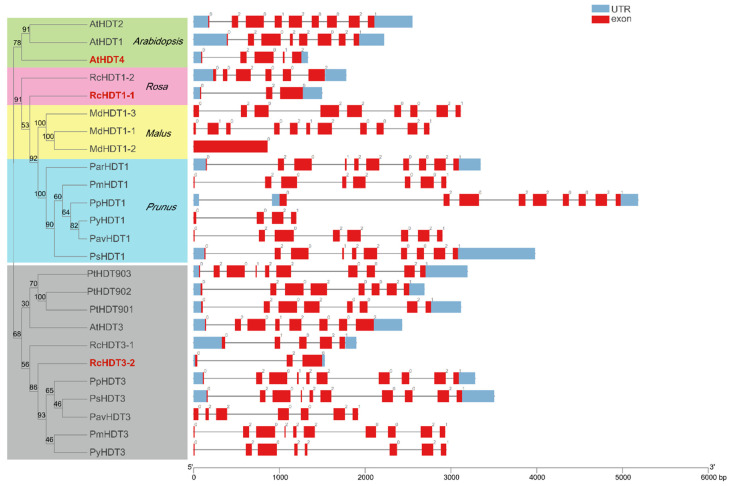
Phylogenetic tree and exons distribution of *HD2* family genes. The unrooted maximum likelihood tree was constructed by IQtree 2.1.3 using complete protein sequences with 1000 bootstraps. The red members represent HD2s that do not contain the zinc-finger domain.

Unlike *HDA1* superfamily, *SIR2* family consists of only two members in most Rosaceae species, namely *SIR1* and *SIR2*. Three members were identified in apple and rose but no *SIR2* genes in Somei-Yoshino (Table 1). For plants specific *HD2* family, only rose has four members corresponding to *Arabidopsis*, other Rosaceae plants have one, two, or three members. The phylogenetic tree of 13 PmHDACs and 18 AtHDACs proteins infers that all the 31 prospective proteins have a great bootstrap value showing the evolutionary relationships between mei and *Arabidopsis* HDACs proteins (Figure 2a). In our results, the phylogenetic trees of *HDACs* (Figure 1 and Figure 3) can also highlight the evolutionary relationship of Rosaceae plants, indicating that *PmHDACs* are more closely related to *Prunus* species like apricot and peach, which corresponds to the previous study [42,43,44].

### 2.3. Gene Structure and Conversed Domains Analysis

To conform all the putative *RoHDACs* gene structures and conversed domains of proteins, we acquire gene exon numbers and positions in the genome as well as motifs and conserved domains of proteins (Figure S5, Table S3). The number of exons in three families exhibit distinct differences, which were ranged from 1 to 24. In the *HDA1* superfamily, the orthologous genes of different species seem to contain a similar exon number. For instance, almost all *RoHDA1* genes have 7 exons and most *RoHDA9* genes have 14 exons. *RoHDA15* and *RoHDA5* genes, containing the average numbers of 16.89 and 14.33 exons, have significantly more exons than *RoHDA8* and *RoHDA14* (4.0 and 7.31) in the Class II group. In the plant-specific HD2 family, most members contain 8 or 10 exons, with an average of 7.52. We identified 8 and 10 exons in *PmHDT1* and *PmHDT3*, respectively (Figure 3). It indicates that the distribution of exons in most HD2 genes seems to reveal a relatively stable evolutionary trend except some short paralogous genes. Besides, Gr2 group members (*AtHDT4, RcHDT1-1, RcHDT3-2*) contain less exons with 3-5. We also found that exon numbers among the paralogous genes, such as *PmHDA8s*, *RcHDA6s*, and *RcHDA14s* are dramatically different in terms of sequence size (Figure S5, Table S3).

**Figure 4:**
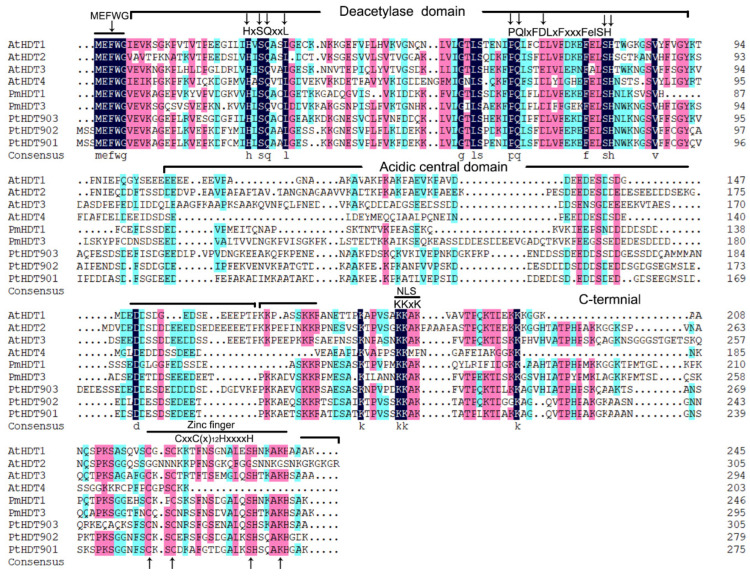
Sequences alignment of HD2 family proteins in Arabidopsis, Populus trichocarpa and mei. Five domains, MEFWG motif, deacetylase domain, acidic central domain, NLS site -KKxK-, and zinc finger domain were marked in these sequences.

Conserved motifs and domains are important components shared by members of gene family as well as the main regions playing functions. In this study, we identified 20 motifs, ranging from 21–80 aa, in all 154 HDACs proteins of 10 plants. It indicates that both the type and the number of motifs in these three types of HDACs are obviously diverse (Figure S5, Table S3). Almost all Class I type proteins have 9 kinds of motifs (motif7,14,10,4,9,5,1,2, and 11). On the contrary, in the class II, the distribution of motif types in HDA5, HDA15, HDA8, and HDA14 clades are slightly diverse. Class IV contains 4 motifs and the SIR2 family contains 3, where motif 16 and 17 are shared both in SRT1 and SRT2, but motif 18 is specific in the SRT2 proteins. We identified only one motif in the HD2 family. Furthermore, we predict the number and the distribution of completely conserved domains in each PmHDACs protein (Figure 2c). Over 8 HDA1 type proteins have a highly characteristic histone deacetylase domain that ranges from 140 to 348 aa, and 2 SIR2-type HDACs have a well conserved Sir2 domain. Moreover, sequence alignment indicates that two HD2 proteins (PmHDT1 and PmHDT3) contain 5 conserved domains: an N-terminal MEFWG- motif, followed by an around 86aa deacetylase catalytic domain, a long highly-variable acidic central domain, a 4 amino acid -KKAK- NLS, and a 22aa C2H2 zinc finger domain in the C-terminal end (Figure 2c and Figure 4). Previse study indicates that the HD2 family are classified into two groups: Gr1 and Gr2 [45]. We propose to define PmHDT1 and PmHDT3 as the Gr1 group based on the zinc finger they have. These domains play the important roles in the function and the classification of HD2 proteins [45].

### 2.4. RoHDACs Gene Locations on Chromosome, Segmental Duplications and Synteny Analysis

The positions of overall 108 *RoHDAC* genes on the chromosome are determined by their genomic distributions, except for 13 *PyHDAC* genes that fail to be assembled into the chromosomal level. The results show that 12 of 13 *PmHDAC* genes are unevenly distributed on 8 chromosomes (Figure 2b). These are the largest three members on chromosomes one and three, respectively, and two genes on chromosome seven. Only one gene is found on chromosomes five and six, and no *PmHDAC* genes are found on the chromosome four. Besides, the location of other *RoHDACs* genes on the chromosome can be found in the Figures S6 and S7. 

**Figure 5:**
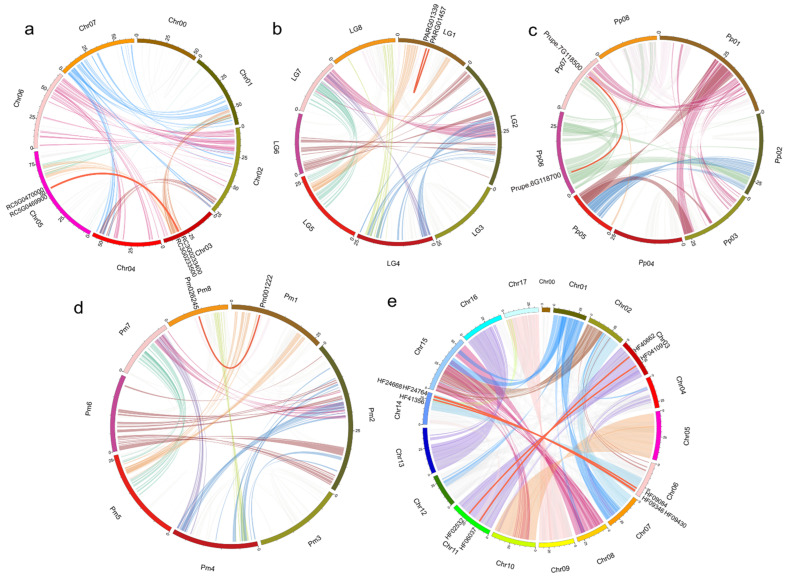
Collinearity of segmental duplication gene pairs of *HDACs* in five Rosaceae species. (**a**) *R. chinensis* (**b**) *P. armeniaca* (**c**) *P. persica* (**d**) *P. mume* (**e**) *M. domestica*. The red lines represent the segment duplication (SD) gene pairs of the *HDACs*.

For TD and SD events, we firstly conducted a genomic comparison among Rosaceae species to identify the syntenic blocks and tandem duplications. Orthologous gene pairs of *HDACs* indicate that there are no TD events but SD events in *RoHDACs* genes. We investigated a total of 11 SD gene pairs in 6 HDACs families, among which 5 gene pairs belonged to *MdHDACs*, 2 belonged to *RcHDACs*, and 1 belonged to *PmHDACs*, *ParHDACs*, *PavHDACs,* and *PpHDACs* (Figure 5, Table S5). It is worth noting that over a half of the SD gene pairs occur in *HD2* families involved in five species, corresponding to the previous study that the *HD2* family expands via several rounds of successive duplication [45]. In mei, only the *Pm001222* and the *Pm026245*, located on chromosomes one and eight, respectively, have a collinearity relationship. However, there are mostly five SD gene pairs in apple *HDACs*, distributed in all three families (Table S4). Previous study showed that a recent whole-genome duplication (WGD) shaped the genome of the domesticated apple, and we compared these five SD gene pairs that belong to duplication regions of the chromosome [42,46], which indicates that the WGD event may mainly contribute to the expansion of the *MdHDACs* family, resulting in the expansion of paralogous genes.

**Figure 6:**
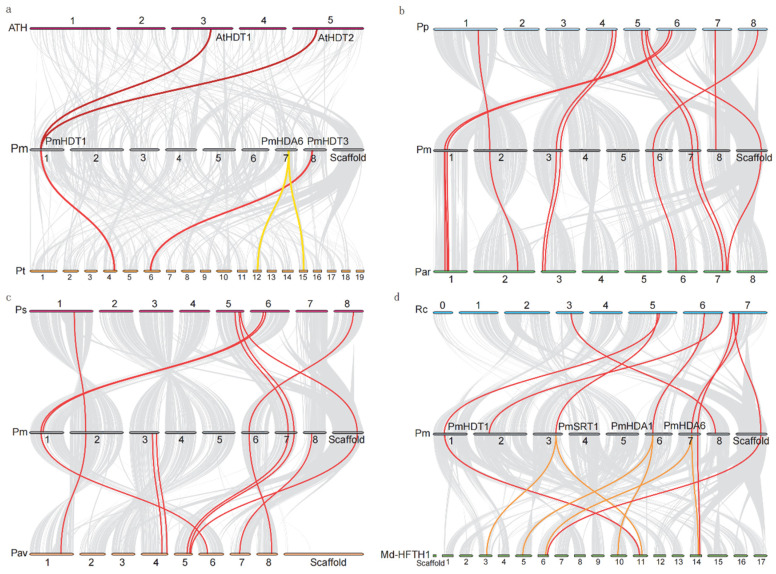
Macrosynteny pattern between the *P. mume* and other plants karyotype of *HDACs.* Grey lines highlight the syntenic blocks spanning the genome. Red, yellow, and orange lines highlight the major segmental duplications of *HDACs* inter-chromosomes. (**a**) *A. thaliana* (ATH) vs. *P. mume* (Pm) vs. *P. trichocarpa* (**b**) *P. persica* (Pp) vs. *P. mume* (Pm) vs. *P. armeniaca* (**c**) *P. salicina* (Ps) vs. *P. mume* (Pm) vs. *P. avium* (Pav) (**d**) *R. chinensis* (Rc) vs. *P. mume* (Pm) vs. *M. domestica (Md-HFTH1)*.

In addition, to further confirm the evolution of Rosacea plants, we conducted intergenomic comparisons and synteny analysis against *Arabidopsis*, *P. trichocarpa*, mei and other Rosacea plants. Only two and four *HDACs* orthologous gene pairs are identified between *Arabidopsis* and mei, as well as *P. trichocarpa* and mei, respectively (Figure 6a). Among them, *PmHDT1* (*Pm001222*), located on chromosome one, has two collinearities (*AtHDT1/2*) in *Arabidopsis,* whereas only one homology gene in other Rosaceas, suggesting that HD2 family genes contracted in some Rosacea genomes. However, we found at least eight orthologous gene pairs among mei and other Rosaceae plants (Figure 6b,c), suggesting that *HDACs* among Rosaceae plants have significantly richer homologous relationships than those in model plants. In addition, there are more chromosomal synchronization collinearity genes between mei and apricot, indicating that they have a very closely relationship. Synteny between mei and apple shows that some *PmHDAC* genes (*PmHDA1/6, PmSRT1)* have two homologies in apple, suggesting the *HDACs* gene family expanded in the apple genome. In summary, it is obvious that the members of the *HDACs* gene family expanded or contracted significantly in various species during the evolution of Rosaceae, which may be related to the genome duplication event [42].

### 2.5. Cis-Element of PmHDACs

**Figure 7:**
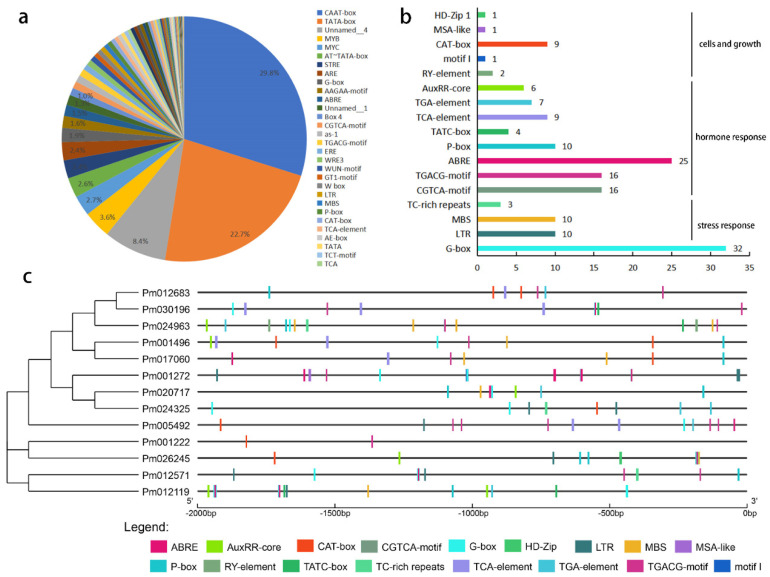
*Cis*−elements analysis of *PmHDAC* gene promoters located on the 2 kb sequences. (**a**) The proportion of all *cis*−elements predicted in the promoters of *PmHDACs* using PlantCARE website. (**b**) Numbers of the *cis*−elements involved in light responsiveness, stresses responsiveness, hormone responsiveness and cells and tissues development (**c**) The distribution of the main 17 *cis*−elements in (**b**) in *PmHDAC* gene promoters.

To confirm the potential regulatory mechanisms of *PmHDACs* in the plant development process, we analyzed the *cis*-element located on the 2.0 kb of all *PmHDAC* genes upstream using the PlantCARE website. Bioinformatics analysis showed that the TATA box and the CAAT-box, defined as common *cis*-acting elements in promoter and enhancer regions, are still the widely distrusted elements (52.5%) (Figure 7a). Then, we selected 17 *cis*-elements related to light, plant hormones, stress response, and the meristem development processes (Table S6). G-box elements, involved in plant light responsiveness, are widely distributed in *PmHDACs* promoters (Figure 7b,c). Notably, some *cis*-elements that have been confirmed as involved in biotic and abiotic stress, such as LTR, MBS, and TC-rich repeats, were comprised in *PmHDACs* (Table S5, Figure 7b). We found that 6 of 13 *PmHDAC* genes have LTR elements: *PmHDA9* contains 3 LTRs, *PmHDA6* and *PmSRT2* have 2 LTRs, and *PmHDA2*, *PmHDT3,* and *PmSRT1* have only one LTR (Figure 7c). These *cis*-elements identified related to hormones and plant stress processes may play an important role in plant resistance to environmental changes. Besides, we found a large number of hormone-related elements such as ABRE, CGTCA-motif, TCACG-motif, P-box, TCA-element, TGA-element, AuxRR-core, and TATC-box that participate in hormonal responses (i.e., gibberellin, abscisic acid, MeJA, auxin, and salicylic acid). 

### 2.6. PmHDACs Expression Profiles in Tissues and Treatments

**Figure 8:**
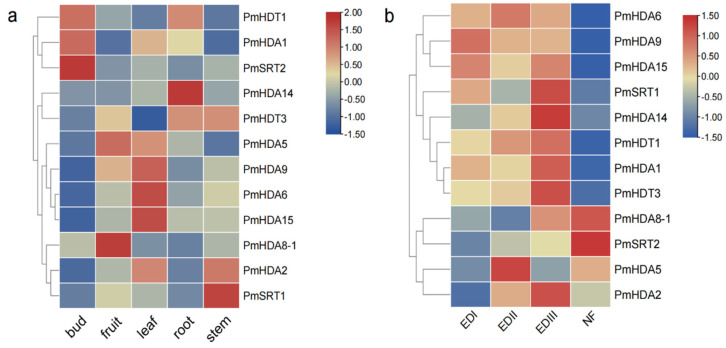
Expression profiles of *PmHDACs*. (**a**) The mRNA accumulation patterns of *PmHDACs* in flower buds, fruits, leaves, roots, and stems tissues of mei. (**b**) *PmHDACs* expressions in flower buds of P. mume cultivar ‘Lve’ under low temperature in three endodormancy stages EDI (Endodormancy I, 0% flush rate), EDII (Endodormancy II, 45% flush rate), EDIII (Endodormancy III, 100% flush rate), and NF stage (Natural Flush, flower buds with green tips and dormancy completely released). The legend represents the FPKM values of gene expressions from RNA−seq after normalized with row scale method.

*PmHDACs* genes are differentially expressed in flower buds, fruits, leaves, roots, and stems, especially genes *PmSRT2*, *PmHDA14*, *PmHDA6*, *PmHDA15*, *PmHDA8-1*, *PmSRT1* are tissue-specific (Figure 8a). For example, *PmSRT2* is expressed in flowering buds, but it has a relatively low expression in other tissues. *PmHDA14* and *PmHDA8-1* are specifically expressed in roots and fruits, respectively. All *PmHDACs* genes show two expression trends during the flowering bud dormancy process (Figure 8b). Four genes (*PmHDA14*, *PmHDT1*, *PmHDT3*, and *PmHDA2*) are gradually up-regulated from EDI to EDIII and up to the peak in the EDIII stage, then abruptly decrease in the fourth NF stage, particularly *PmHDA14* and *PmHDT3*. The expression of *PmHDA6/9/15* genes is stably high in three endodormancy stages and also decreases in the NF stage. *PmSRT1* gene is highly expressed in EDII. On the contrary, the expression of *PmHDA8-1* and *PmSRT2* gradually accumulates and reaches a peak in the NF stage when the bud dormancy is released. These expression patterns suggest that *PmHDACs* may be involved in the bud dormancy release process by deacetylating the target genes of different signaling pathways at different development stages.

**Figure 9:**
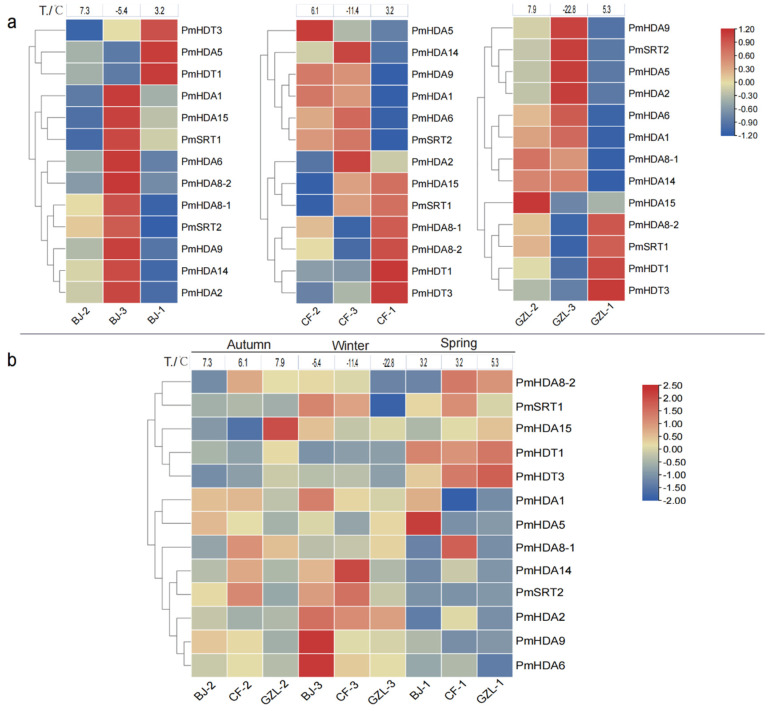
Expression accumulation in stems of cold−insensitive cultivar ‘Songchun’ under three natural low temperatures (1, 2, 3) at three test sites: Beijing (BJ), Chifeng (CF), and Gongzhuling (GZL). (**a**) Expression comparison profiles of *PmHDACs* in different seasons at the same test site. (**b**) The expressions of *PmHDACs* in different test sites. The numbers up−on the heatmap represent the temperature in this stage. In three temperatures, the 2nd stage was the autumn deciduous period, the 3rd was the winter bud dormancy period, in which the temperature is the lowest, and the 1st was germination period in early spring. Cold acclimation period began with the 2nd to 3rd stages and lost at the 3rd to 1st stage. The legend represents the FPKM values of gene expressions from RNA−seq after they were normalized with the row scale method.

We also detect the expression profiles in stems of cold-insensitive cultivar ‘Songchun’ under three low temperatures at three test sites: Beijing (BJ), Chifeng (CF), and Gongzhuling (GZL) (Figure 9). Some of *PmHDACs* genes show similar expression patterns during the change of temperature in different phenological periods at the same latitude test site, respectively (Figure 9a). For example, at the Beijing site, a total of 10 genes (*PmHDA1/6/9/15/2/14/8-1/8-2*, *PmSRT1/2*) show up-regulation in autumn and in winter (2nd to 3rd stages) but then decrease in the early-spring (1st stage). However, the expression levels of *PmHDA5* and *PmHDT1* genes are down-regulated from the 2nd to the 3rd stages and then increase in the first stage. We find the gene expression trend of Gongzhuling and Chifeng sites are relatively consistent with that in Beijing. In summary, we find *PmHDA1/6/9* and *PmSRT2* genes are all up-regulated at the cold acclimation period (2 to 3), then down-regulated at the cold acclimation lost period (3 to 1) in three sites, while *PmHDT1/3* has the opposite expression profiles, indicating that these genes are positively or negatively correlated with low temperature response. However, the expressions of most *PmHDACs* in the same season have slight differences at the different test sites, among which some genes like *PmHDA15* are highly expressed at the GZL site in autumn, *PmHDA6/9* are highly expressed at the BJ site, while *PmHDA14* shows high expressions at the GZL site in winter. More than half of genes have a relatively low expression in spring, except *PmHDT1/3* (Figure 9b). These results indicate that the expression diversities of *PmHDACs* in different sites may be caused by geographical environmental factors or cold-resistant differences of plant individuals.

### 2.7. qRT-PCR of PmHDACs under Cold Stress and Gene Functional Annotation

**Figure 10:**
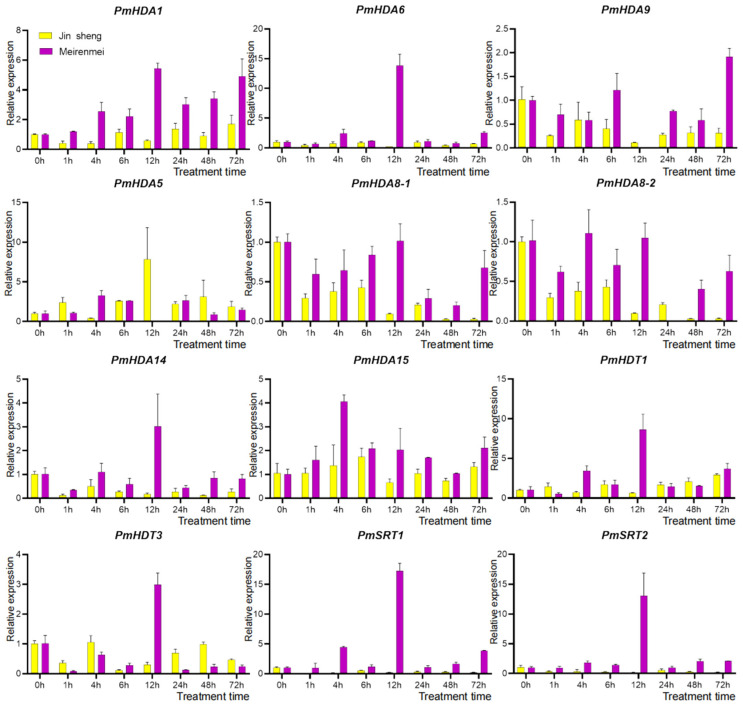
qRT-PCR analysis of *PmHDACs* in annual branch of cold-sensitive cultivar ‘Jinsheng’ and cold-insensitive cultivar ‘Meirenmei’ under low temperature treatment. Cold treatments were under 4 °C at 0, 1, 4, 6, 12, 24, 48 and 72 h. The yellow and purple represent cold-sensitive cultivar ‘Jinsheng’ and cold-insensitive cultivar ‘Meirenmei’, respectively.

To further confirm the putative roles of *PmHDACs* genes in response to cold stress in mei, the expression levels of *PmHDACs* under 4 °C low temperature treatment are determined by qRT-PCR in cold-sensitive cultivar ‘Jinsheng’ and cold-insensitive cultivar ‘Meirenmei’ (Figure 10). All *PmHDACs* members show significant expression profiles under cold treatments in the two cultivars. Obviously, most of the genes are induced with higher expression levels in ‘Meirenmei’, by contrast, repressed in the cold sensitive cultivar ‘Jinsheng’. For instance, *PmHDA6*, *PmHDA14*, *PmHDT1*, *PmHDT3*, *PmSRT1*, and *PmSRT2* genes are significantly induced and highly expressed at 12 h under cold, indicating that they might be involved in the cold stress response process of ‘Meirenmei’ at this stage. In addition, the expression of the *PmHDA1* gene is markedly up-regulated at 1 h to 72 h under cold treatment compared without cold (0 h), also peaking at 12 h. These above genes may have a close positive correlation with cold tolerance of ‘Meirenmei’, all of which are induced by low-temperature, and are likely to be involved in the process of cold stress regulation by modifying low temperature sensitive key genes through deacetylation. Importantly, we find that genes *PmHDA6*, *PmHDA9*, *PmHDA8-1/8-2*, *PmHDA14*, *PmSRT1*, and *PmSRT2* are strongly down-regulated in ‘Jinsheng’: their expressions are barely detectable in the later cold treatment stages (12–72 h), which indicates that these genes are also likely to be negatively involved in the cold intolerance of ‘Jinsheng’. The *PmHDA5* gene is the only member highly expressed in the ‘Jinsheng’, and the expression peak also occurs at 12 h after cold treatment, indicating that it may be positively involved in the regulation of the cold intolerance in ‘Jinsheng’. Thus, these expression results demonstrate that *HDACs* genes show the functional diversity of a cold stress response and the similarity in functional mechanisms. All of them mainly enrich in protein deacetylation, chromosome organization, and other epigenetic processes, and they may target activated or inhibitive genes by deacetylation modification to participate in gene silencing regulation, thereby positively or negatively regulating mei cold sensory processes (Figure S8).

## 3. Discussion

The essential histone deacetylation genes *HDACs* can interact with other transcription factors to silence gene expression through deacetylation modification [47]. They are widely involved in plant vegetative and reproductive growth processes, seed maturation and stress responses [15,48]. In this study, we have identified a total of 121 *RoHDACs* genes in 8 Rosaceae genomes, which are classified into 3 families (*RPD3/HDA1*, *HD2* and *SIR2*). For the *HDA1* superfamily, all 12 *AtHDA1*-type genes are initially divided into classes I, II, and III [13] of which, *AtHDA8*, *AtHDA14*, *AtHDA10*, and *AtHDA17* are still unclassified into any class genes [13,48]. Later research indicates that *AtHDA8* and *AtHDA14* are classified into Class II and three clusters (Class I, II, and IV) confirmed [12]. However, the rice HDA1 proteins are divided into four classes [14]. Our study suggests that the orthologues genes of *HDA8* and *HDA14* are clustered as class II proteins with a high bootstrap value (Figure 1) as well as shared at least five motifs (Figure S5), which corresponds to the later classification of *Arabidopsis HDA1* [12]. This study also indicates that each of the three *HDACs* subfamilies has its own conserved domains. It is worth noting that the plant-specific HD2 family contains five conserved domains, of which the N-terminal motif MEFWG-, important for the gene regulation activity of HDs [49], is also highly conserved among most of the Rosaceae *HDs* identified (Figure 2 and Figure S9), which is consistent with the previous research [45]. Besides, previous study shows that the length of almost all HD2 protein differences is strongly correlated with the length of the highly-variable acidic central domain [45]. Sequence alignment shows that PmHDT1/3 acidic central domains are relatively less conserved, which is probably the main reason for the differences in gene functions.

Gene duplication events are considered to be the major forms of gene family expansion in the history of gene evolution [50]. In our study, a total of 11 SD gene pairs were identified in 8 Rosacea HDACs. Previous phylogenetic research confirms that gene duplication events or sequence rearrangements occur successively during the evolution of the *Arabidopsis HDA1* superfamily, resulting in an increase in gene members (*AtHDA10/17/18*) [13,51]. Notably, similar homologous gene duplication and rearrangement events also occur in eight Rosaceae *HDA1* superfamily, leading to an uneven distribution of HDA1 genes in different species. Having the most numbers of *HDACs*, a total of 21 genes are identified in the apple genome. So why are there significantly more *HDACs* in apples than in the *Prunus* species? Firstly, some paralogous genes are searched in *MdHDACs*, where *MdHDA1* has three paralogous genes (Figure S3) and *MdHDA5/6/15* have two. These homologous genes pairs are found at the same time as the SD gene pairs (consistent with WGD blocks [42]) in apple genome (Table S4). For the collinearity results in apple, not only *HDA1* family but also the *SIR2* and the *HD2* genes have obvious SD events, which further indicates that the recent WGD event and the increase in chromosomes as an ancient tetraploid plant lead to the significant expansion of the apple *HDACs* family [42]. Besides, we found that most *HDA8* genes of the *HDA1* family in Rosaceae plants contain two paralogous genes (Table S3). For instant, PmHDA8-1 protein is highly similar with the PmHDA8-2 (Figure S1). Similar gene expansions are observed in other *RoHDACs* like *PyHDA14* and *RcHDAC14* genes (Figures S2 and S4). In our research, the SD gene pairs of *RoHDACs* exist in most *HD2* family members, such as *PmHDT1* and *PmHDAT3* (Table S4), demonstrating that the HD2 family may have expanded via several rounds of successive duplication [45]. Obviously, we find that some HD2 proteins lost almost all of the C-terminal region, including the C2H2 zinc finger domain, such as RcHDT1-1 and RcHDT3-2 that can be defined as the Gr2 group (Figure S9). However, the sequences of these two Gr2 HD2s are conserved with those of Gr1 members, and it is reasonable to postulate that Gr2 evolves from Gr1 gene [45]. 

Epigenetic modification control plant cold responses [52]. In *Arabidopsis*, *HDA6* is one of the *HDAC* genes analyzed that plays a key role in cold tolerance. The mutant *axe1-5* shows reduced freezing tolerance compared with the wild-type plants under cold treatment, indicating that *HDA6* plays a critical role in regulating the cold acclimation process that confers freezing resistance in *Arabidopsis* [36]. In our research, three cold stress treatments were analyzed in different mei cultivars and tissues. Based on the above results, we also obtained some genes closely related to cold stress. First, the important gene *PmHDA6* responded to cold stress in both flower buds and stems at different sites (Figure 8 and Figure 9). Furthermore, qRT-PCR results indicate that the expression levels of *PmHDA6* are strongly up-regulated at 12 h under 4 °C cold treatments, then abruptly reduce later in cold tolerance cultivar ‘Meirenmei’. Besides, the expression levels of *PmHDA1* gene in ‘Meirenmei’ are strongly increased during the cold stress treatment, which is quite similar to the expression of *AtHDA6* [36]. These results suggest that *PmHDA1* and *PmHDA6*, as hub genes, are probably involved in the cold response to promote the freezing resistance of ‘Meirenmei’ through regulation of cold-related genes. Other *PmHDA1* genes *PmHDA9*, *PmHDA8-1/8-2*, *PmHDA14*, *PmHDA5*, and *PmHDA2* are also significantly negatively or positively responsive to cold stress in two cultivars. In banana, MaMYB4 recruits MaHDA2 to modulate mechanisms of fatty acid biosynthesis during a cold stress response in fruits [53]. Except for the cold stress, *HDA1* genes play important roles in other abiotic stress through forming protein complex with TFs, such as WRKY and hormone signals [32,33,34,54,55]. The *Arabidopsis* HOS15, a WD40-repeat protein, interacts with HDACs to regulate the plant development process [56,57], particularly, functions as a repressor to control cold stress-regulated gene expression through chromatin modification [58,59]. HD2 proteins also play important roles in abiotic stress responses, including cold [41,59,60,61]. AtHD2C (AtHDT3) interacts with HOS15, associated with cold signaling gene COR (COLD RESPONSIVE) and CBF (C-REPEAT (CRT) BINDING FACTOR) in response to cold stress [58]. Recent study indicates that HD2 proteins can interact with HDA1-type histone deacetylases, such as HDA6 and HDA9 [38,40]. In mei, the expression accumulation of *PmHD2* genes is detected in stems and flower buds under cold treatment. Among them, *PmHDT1* is highly expressed in ‘Meirenmei’ under cold treatment, which is consistent with *PmHDA6* (Figure 10). Collectively, we infer that PmHDACs may function as the similar protein complex to be involved in cold stress in mei plant. 

Additionally, it is worthy to note that mei *SIR2* genes are strongly up-regulated in ‘Meirenmei’ cultivar while down-regulated in ‘Jinsheng’, which shows the significant cultivar association. We find that the motif 18 that is highly conserved only consist in the SRT2 proteins but not in the SRT1 (Figure S10). Furthermore, the sequence conservation of SRT1 proteins or SRT2 proteins are significantly higher than that between the two proteins (Figure S10). It suggests that *PmSRT1* and *PmSRT2* may be involved in response to cold stress with different regulatory mechanisms in different cultivars, although there is little function data available about plant *SIR2*-type *HDACs* [48]. Combined with *cis*-elements identified in *PmHDACs* (Figure 7), we find that almost a half of genes contain the low temperature response element LTR, which have been detected to bind to transcript factors like bHLH to regulate plant development [62,63,64]. In summary, our results confirm that histone deacetylases *PmHDACs* play important roles in the regulation of the abiotic stress response regarding the cold tolerance of mei in north China. However, how their interactions form the protein complex that regulates those genes related to cold stress and affect the cold resistance of mei requires further research in the future.

## 4. Materials and Methods

### 4.1. Plants Genome Resources

The high-quality genome assembly and annotation files of *A. thaliana* (TAIR10.41), *P. trichocarpa* (v4.0), *P. mume* (v1.0), *P. salicina* ‘Sanyueli’ (v2.0), *P. armeniaca* (v1.0), *P. persica* ‘Lovell’ (v2.0), *P. avium* (v1.0.a1), *P. yedoensis* var. nudiflora (v1.0), *M. domenstica* ‘HFTH1′ (v1.0), and *R. chinensis* ‘Old Blush’ (v1.0) were download from the Genome Database for Rosaceae (GDR, https://www.rosaceae.org, accessed on 5 July 2021) [65] and from Phytozome v12.1 (https://phytozome.jgi.doe.gov/pz/portal.html, accessed on 5 July 2021) [66].

### 4.2. Identification of HDACs Gene Family

The HMM search 2.0 was used to identified the *HDA1* and the *SIR2* families with a histone deacetylase domain and a SIR2 domain (PF00850, PF02146) from the Pfam database v32.0 [67] (http://pfam.xfam.org, accessed on 7 July 2021). *HD2* genes were identified by BLASTP according to the *Arabidopsis HD2* proteins. After proofreading using a phylogenetic tree, sequence size, and conserved domains, we deleted the wrong gene members. Ultimately, the *HDACs* members of 8 Rosaceae plants were acquired.

According to gene numbers and conserved domains, transmembrane domains and signal peptides were acquired on the SMART v9.0 [68] (http://smart.embl-heidelberg.de, accessed on 23 July 2021), TMHMM-2.0 [69] (https://services.healthtech.dtu.dk/service.php?TMHMM-2.0, accessed on 6 August 2021), and SignalP-5.0 [70] (https://services.healthtech.dtu.dk/service.php?SignalP-5.0, accessed on 6 August 2021) websites. The NLS and the physical and chemical parameters of proteins were predicted on the WoLF PSORT (https://wolfpsort.hgc.jp/, accessed on 6 August 2021) and ProParam (https://web.expasy.org/protparam/, accessed on 7 August 2021).

### 4.3. Phylogenetic Analysis and Classification of HDACs Genes

With identified *HDACs*, we first conducted a codon align of mei and *Arabidopsis* protein sequences. Using a Muscle method, neighbor-joining trees were constructed by MEGA 7.0 [71]. To obtain the maximum-likelihood tree, we generated a set of protein multiple sequence alignments of HDACs proteins with MAFFT v7 [72] (https://mafft.cbrc.jp/alignment/server/, accessed on 10 August 2021), and we constructed the phylogenetic trees using IQtree 2.1.3 [73] (http://iqtree.cibiv.univie.ac.at/, accessed on 10 August 2021). For the putative name of Rosaceae *HDACs* genes, we divided all the Rosaceae *HDACs* into subgroups and named the homologous gene based on *Arabidopsis HDACs* genes.

### 4.4. Gene Structure and Protein Conserved Motif Analysis

We obtained the exons, introns, and UTR (untranslated Region) location information of the genes from the genome annotation file. The RoHDACs proteins were submitted to MEME-v4.12.0 [74] with settings: -maxsize 6,000,000 -mod anr -nmotifs 20 -minw 6 -maxw 100 to search for conversed motifs. Finally, TBtools [75] was used to conduct the tree-structure-motif map.

### 4.5. Duplications, Synteny and Genes Chromosome Location Analysis

Protein sequences of each Rosaceae specie were used to makeblastb and blastp all-vs-all with Blast 2.6 software, respectively. We analyzed eight Rosaceae WGD events by McscanX [76] with default settings. The segmental duplication (SD) gene pairs and tandem duplication (TD) events of RoHDACs were acquired from synteny blocks and the tandem gene pairs. Synteny circle-map were plotted by CIRCOS [77]. The collinearity of HDACs in different genomes was analyzed by McscanX as above. Chromosomal location of RoHDACs and total length of chromosomes were extracted from a genome annotation gff file and a genome sequence, and they were marked by MG2C_v2.0 (http://mg2c.iask.in/mg2c_v2.0/, accessed on 13 August 2021) [78].

### 4.6. PmHDACs Cis-Acting Element Analysis

We obtained the 2000 bp promoter sequences upstream of the *PmHDACs* gene members, and we submitted it on the website plantCARE [79] to analyze *cis*-acting elements they contained; then, we constructed the element distribution with GSDS3.0 (http://gsds.gao-lab.org, accessed on 17 August 2021). 

### 4.7. Expression Profiles of PmHDACs

To investigate the potential functions of *PmHDACs* in different tissues and cold stress response, RNA-seq data of five different tissues of mei (flower buds, fruits, leaves, roots, and stems) [80] and flower buds of mei ‘Lve’ cultivar exposed in three dormancy status EDI (Endodormancy I, 0% flush rate), EDII (Endodormancy II, 45% flush rate), EDIII (Endodormancy III, 100% flush rate), and NF stage (Natural Flush) [81] were obtained. The expression profiles were also analyzed in RNA-seq data of ‘Songchun’ cultivar stems at three different geographical sites: Beijing (BJ, 54′39° N, 28′116° E), Chifeng (CF, 17′42° N, 58′118° E), and Gongzhuling (GZL, 42′43° N, 47′124° E) in three phenological stages (autumn deciduous initiation, winter dormancy, and spring germination) under natural cold. 

The heatmap of gene expression was mapped by FPKM values using TBtools [75] with a normalized row scale and row cluster method.

### 4.8. Plant Materials, Cold Stress Treatments and qRT-PCR

Branches of cold-sensitive cultivar ‘Jinsheng’ and cold-tolerated cultivar ‘Meirenmei’ were collected from Jiufeng International Plum Blossom Garden, Beijing, China (07′40° N, 11′116° E). Plants were maintained in water overnight at 22 °C and treated under 4 °C with 1, 3, 6 12, 24, 36, 48, and 72 h under long-day conditions (16-h light/8-h dark). Then we obtained annul branches to extract total RNA.

Total RNA was isolated by an RNAprep Pure Plant Plus Kit (Qiagen, Beijing, China) and then synthesized with cDNA using the ReverTra Ace®qPCR RT Master Mix with gDNA Remover (Toyobo, Osaka, Japan). A quantitative RT-PCR was performed on a qTOWER2.2 System (analytikjena, Jena, German), using a SYBR®Green Premix Pro Taq HS qPCR Kit (AccurateBiology, Hunan, China). The reaction system was a total of 20 μL with a 10 μL SYBR®Green Premix Pro Taq HS qPCR Kit, 8 μL 10× forward and reverse primers mix, and 2 μL 10× cDNA samples. The reaction program was set as follows: an initial denaturation step (30 s at 95 °C), followed by 40 cycles of 5 s at 95 °C, 30 s at 50–60 °C, and 30 s 72 °C. The relative expression levels of the genes were calculated using the ∆∆Ct method and normalized using the *PmActin* reference gene. The primers used in this study were listed in Table S7.

### 4.9. GO Annotation and Enrichment Analysis

GO annotation data of *PmHDACs* genes were extracted from mei genome GO annotation files (background) and then submitted on the website omicshare (https://www.omicshare.com/, accessed on 13 September 2021) to perform further GO enrichment analysis.

## 5. Conclusions

In this study, a total of 121 *HDACs* are identified in 8 Rosaceae plants, among which 13 genes are from woody plant mei. The detailed genome-wide characterization analyses of *HDACs* are first confirmed in Rosaceae plants, including phylogenetic evolution, subfamily classification, gene structure, conserved domain, gene locations, and SD events. Besides, we focus on the expression profiles of *HDACs* in mei. The mRNA accumulation levels of *PmHDACs* in mei various tissues reveal relatively tissue-specific or wide expressions profiles. RNA-seq data and qRT-PCR experiment in cold treatment suggest that *PmHDAC* genes significantly respond to cold stress with up or down regulated expression pattens. Our research provides an insight into the phylogenetic relationship of the *HDACs* family in Rosaceaes, and it functions as an investigation of *PmHDACs* response to cold stress in mei. 

## Figures and Tables

**Table 1:** *HDACs* genes identified in *HDACs* family of 10 plants.

Subfamily	*A.thaliana*	*P.trichocarpa*	*P.mume*	*P.armeniaca*	*P.salicina*	*P.persica*	*P.avium*	*P.yedoensis*	*M.domenstica*	*R.chinensis*
HDA1/Class I	5	5	3	3	3	3	4	4	8	4
HDA1/Class II	6	4	5	5	5	5	7	5	6	7
HDA1/Class IV	1	1	1	1	1	1	1	2	1	2
HD2	4	3	2	1	2	2	2	2	3	4
SIR2	2	2	2	2	2	2	2	/	3	3
Total numbers	18	15	13	12	13	13	16	13	21	20

## Data Availability

The data are included into supplementary materials.

## Supplementary Materials

The following supporting information can be downloaded: at https://www.mdpi.com/article/10.3390/ijms23115957/s1.
