# Supplementary material for: HDACs Gene Family Analysis of Eight Rosaceae Genomes Reveals the Genomic Marker of Cold Stress in Prunus mume
Source: Int J Mol Sci. 2022 May 25;23(11):5957. doi: 10.3390/ijms23115957 (PMC9180812; doi:10.3390/ijms23115957)
Supplement: Supplementary file 1 [file ijms-23-05957-s001.zip › ijms-1723682-supplementary/Supplementary Materials/Figure S1-4,6-10.pdf]

```
AtHDA8_AT1G08460.1_   .......MVTNRVDVFWHEGMLRHDAVEGVFDTGYDPGFLDVLEKHPENADRVRNMLSIL    53
Pm001496              MAASTATAPTDRIDVFWDSGMLNHNSGNGVFDTGMDPGFLDVLEKHPENSDRVKNMVSIL    60
Pm017060              MAASTATAPTDRIDVFWDSGMLNHNSGNGVFDTGMDPGFLDVLEKHPENSDRVKNMVSIL    60
Consensus                      t r dvfw  gml h    gvfdtg dpgfldvlekhpen drv nm sil

AtHDA8_AT1G08460.1_   RRGPIAPHVNWFTGLPAIVSELLMFHTSEYIEKLVEADKSGERCEIAAGTFMSPGSWEAA   113
Pm001496              KRGPISPYISWLPGRPMLLSELLSFHSSEYINELVEADRDGGK.MLCSGTFLNPGSWDAS   119
Pm017060              KRGPISPYISWLPGRPMLLSELLSFHSSEYINELVEADRDGGK.MLCSGTFLNPGSWDAS   119
Consensus              rgpi p   w  g p   sell fh seyi  lvead  g       gtf  pgsw a

AtHDA8_AT1G08460.1_   LLAAGTTLSAMQHILDCHGKIAYALVRPPGHHSQPTQADGYCFLNNAALAVKLALNSGSC   173
Pm001496              LLAAGTTLSAMKHVLDGHGKIAYALVRPPGHHAQPTRADGYCFLNNAGLAVQFALNSG.C   178
Pm017060              LLAAAYS..............SRWLLLP................DNAGLAVQLALNSG.C   148
Consensus             llaa                    l  p                 na lav  alnsg c

AtHDA8_AT1G08460.1_   SRVAVIDIDVHYGNGTAEGFYTSDKVLTVSLHMNHGSWGSSHPQKGSIDELGEDVGLGYN   233
Pm001496              AKVAVIDIDVHYGNGTAEGFYRSDKVLTISLHMNHGSWGPSHPQNGSVDELGEAEGFGYN   238
Pm017060              AKVAVIDIDVHYGNGTAERFYRSDKVLTTSLHMHHGSWGPSHPQNGSIDELDEGEGFNYN   208
Consensus               vavididvhygngtae fy sdkvlt slhm hgswg shpq gs del e  g  yn

AtHDA8_AT1G08460.1_   LNVPLPNGTGDRGYEYAMNELVVPAVRRFGPDMVVLVVGQDSSAVSS.........FFAQ   284
Pm001496              LNIPLPNGTGDRGYRHAMTELVVPMIQKFEPEMMVLVIGQDSSAFDPNGRQCLTMEGYQE   298
Pm017060              LNIPLPYGTGDRGYKHDMTELVVPMIQKFEPEMMVLVAGQDSSAGSKGRR....FAQSSD   264
Consensus             ln plp gtgdrgy   m elvvp    f p m vlv gqdssa

AtHDA8_AT1G08460.1_   IR..VDYLTEHLLR............VLFAIIVLVTSKSLMYAAPIWLCCDKSA......   324
Pm001496              IGRIVRSLADRHCSGRLLIVQEGGYHVTYSAYCLHATLEGVLNLPTSLLSDPIAYYPEDE   358
Pm017060              VGLCASCCDDDTCL............LCFRFGSSELRQVVVPSEEHLPLAGRKTCLPWCS   312
Consensus

AtHDA8_AT1G08460.1_   .......................                                         324
Pm001496              ALAVEVIKSIKKFQKDNVPFLKG                                         381
Pm017060              ITLG...................                                         316
Consensus
```

Figure S1 Sequences alignment of HDA8 homologous proteins in *Arabidopsis* and mei.

```
PQP91789.1   MELLTVSFSSSLAGNALFLARQHLCKWKNHSRFDLEAKFVRNRILNKSLRRGRRCLSEKREASISCSTGTGEDPLIPVNK    80
PQP94174.1   ................................................................................     0
PQP91793.1   ................................................................................     0
Consensus

PQP91789.1   KLSDARVIYAVAPAMGHNQESHPESHFRVPAIVNALEKWELSPKFRGLEVIELENFNAASSNDIASVHTRAYVSGLEKAM   160
PQP94174.1   ..............MGHNQESHPESHFRVPAIVNALEKWELSPKFRGLEVIELENFNAASSNDIASVHTRAYVSGLEKAM    66
PQP91793.1   .......................................MANPQTLSIEDSSIDSTQVTLLKNLLEAGRHPLVQ....AM    37
Consensus                                              p     e                       v     am

PQP91789.1   DQASKQGIILIEGSGPTYATVDTFRESLVAAGAGISLIDAVV................AESKISKSPPVGFALIRPPGHH   224
PQP94174.1   DQASKQGIILIEGSGPTYATVDTFRESLVAAGAGIALIDAVV................AESKISKSPPVGFALIRPPGHH   130
PQP91793.1   DQASKQGIILIEGSGPTYATVDTFQESLVAAGAGIALIDAVILFEVSFFNTSEFIARVAESKISKSPPVGFALIRPPGHH   117
Consensus    dqaskqgiiliegsgptyatvdtf eslvaagagi lidav                 aeskisksppvgfalirppghh

PQP91789.1   AIPKGPMGFCVFGNVAVAARYAQRSHGLKRVFIIDFDVHHGNGTNDAFYDDPDIFFLSTHQDGSYPGTGKFEEVGHGDGE   304
PQP94174.1   AIPKGPMGFCVFGNVAVAARYAQRSHGLKRVFIIDFDVHHGNGTNDAFYDDPDIFFLSTHQDGSYPGTGKFEEVGHGDGE   210
PQP91793.1   AIPKGPMGFCVFGNVAVAARYAQHSHGLKRVFIIDFDVHHGNGTNDAFYDDPDIFFLSTHQ..VWIGFGLV.........   186
Consensus    aipkgpmgfcvfgnvavaaryaq shglkrvfiidfdvhhgngtndafyddpdifflsthq     g g

PQP91789.1   GTTLNLPLPGGSGDTAMRTVFDEIIVPCAQRFKPDIILVSAGYDAHVLDPLASLQFTTGTYYMLASYIQQLANDLCGGRC   384
PQP94174.1   GTTLNLPLPGGSGDTAMRTVFDEIIVPCAQRFRPDIILVSAGYDAHVLDPLASLQFTTGTYYMLASYIQQLANDLCGGRC   290
PQP91793.1   ................................................................................   186
Consensus

PQP91789.1   VFFLEGGYNLKSLSYSVADSFRAFLGEPSLASEFDNPAILHEEPSTRVRQAIERVKHIHS                       444
PQP94174.1   VFFLEGGYNLKSLSYSVADSFRAFLGEPSLASEFDNPAILYEEPSTRVRQAIERVKHIHS                       350
PQP91793.1   ............................................................                       186
Consensus
```

Figure S2 Sequences alignment of three PyHDA14 homologous proteins in Somei-Yoshino.

```
HF10663-RA   MDTGGNSLVSGPDGVKRKVSYFYDPEVGNYYYGQGHPMKPHRIRMTHALLAHYGLLQNMQVLKPYPARDRDLCRFHADDY    80
HF36232-RA   MDTGGNSLASGPDGVKRKVSYFYDPEVGNYYYGQGHPMKPHRIRMTHALLAHYGLLQNMQVLKPYPARDRDLCRFHADDY    80
HF10684-RA   MDTGGNSLVSGPDGVKRKVSYFYDPEVGNYYYGQGHPMKPHRIRMTHALLAHYGLLQNMQVLKPYPAR..DLCRFHA...    75
Consensus    mdtggnsl sgpdgvkrkvsyfydpevgnyyygqghpmkphrirmthallahygllqnmqvlkpypar  dlcrfha

HF10663-RA   VAFLRNITPETQQDQLRQLKRFNVGEDCPVFDGLYSFCQTYAGGSVGGAVKLNHGICDISINWAGGLHHAKKCEASGFCY   160
HF36232-RA   VAFLRNITPETQQDQLRQLKRFNVGEDCPVFDGLYSFCQTYAGGSVGGAVKLNHGICDISINWAGGLHHAKKCEASGFCY   160
HF10684-RA   .........ETQQDQLRQLKRFNVGEDCPVFDGLYSFCQTYAGGSVGGAVKLNHGICDISINWAGGLHHAKNCEASGFCY   146
Consensus             etqqdqlrqlkrfnvgedcpvfdglysfcqtyaggsvggavklnhgicdisinwagglhhak ceasgfcy

HF10663-RA   VNDIVLAILELLKQHERVLYVDIDIHHGDGVEEAFYTTDRVMTVSFHKFGDYFPGTGDIRDIGYGKGKYYSLNVPLDDGI   240
HF36232-RA   VNDIVLAILELLKQHERVLYVDIDIHHGDGVEEAFYTTDRVMTVSFHKFGDYFPGTGDIRDIGYGKGKYYSLNVPLDDGI   240
HF10684-RA   VNDIVLAILELLK...................................................................   159
Consensus    vndivlailellk

HF10663-RA   DDESYHYLFKPIIGKVMEIFKPGVVVLQCGADSLSGDRLGCFNLSIKGHAECVRYMRSFNVPLLLLGGGGYTIRNVARCW   320
HF36232-RA   DDESYHYLFKPIIGKVMEIFKPGAVVLQCGADSLSGDRLGCFNLSIKGHAECVRYMRSFNVPLLLLGGGGYTIRNVARCW   320
HF10684-RA   ......................................LGCFNLSIKGHAECVRYMRSFNVPLLLLGGGGYTIRNVARCW   201
Consensus                                          lgcfnlsikghaecvrymrsfnvpllllggggytirnvarcw

HF10663-RA   CYETGVALGAEIEDKMPQHEYYEYFGPDYTLHVAPSNMENKNSHMLLEEIRSKLLENLSRLQHAPSVQFQERPPDTELPE   400
HF36232-RA   CYETGVALGAEIEDKMPQHEYYEYFGPDYTLHVAPSNMENKNSHVLLEEIRSKLLENLSTLQHAPSVQFQDRPPDTELPE   400
HF10684-RA   CYETGVALGAEIEDKMPQHEYYEYFGPDYTLHVAPSNMENKNSHMLLEEIRSKLLENLSRLQHAPSVQFQERPPDTELPE   281
Consensus    cyetgvalgaeiedkmpqheyyeyfgpdytlhvapsnmenknsh lleeirskllenls lqhapsvqfq rppdtelpe

HF10663-RA   ENEEQDDPDERWDPDSDMEVDDERKPLPSRVKKEIVEPEVKDPKGTSENARSSGYDPAVDEITTGAKALDMGSGSVDEPT   480
HF36232-RA   ENEEQDDPDERWDPDSDMEVDDERKPLPSRVKKEIVEPEVKDPKGTSENARSSGYDPAVDEITTGTKALDMGSGSADEPA   480
HF10684-RA   .....DDPDERWDPDSDMEVDDERKPLPSRVKKEIVEPEVKDPKGTSENARSSGYDPAVDEITTGAKALDMGSGSVDEPT   356
Consensus         ddpderwdpdsdmevdderkplpsrvkkeivepevkdpkgtsenarssgydpavdeittg kaldmgsgs dep

HF10663-RA   VKVEQDTMNKPADQ                                                                     494
HF36232-RA   VKVEQETTNKPADQ                                                                     494
HF10684-RA   VKVEQDTMNKPADQ                                                                     370
Consensus    vkveq t nkpadq
```

Figure S3 Sequences alignment of three MdHDA1 homologous proteins in apple.

```
RC2G0299600   MFFLKFGFSLMYHFVYCKDSEDNCSGLGNVLFLARQRFCNWKSPERLHLDAIFATDGILYKNLRRGRGCFSKTTEASSSF    80
RC6G0022900   ................................................................................     0
RC1G0265100   ................................................................................     0
RC6G0533000   ................................................................................     0
Consensus

RC2G0299600   SNGTEESPYIPVNTKPSDARIIYAVAPAMGHNQEAHPESHFRVPAIVQALDKLELTPKFRGSKVIELQNFRPASVDDITS   160
RC6G0022900   ................................................................................     0
RC1G0265100   ................................................................................     0
RC6G0533000   ................................................................................     0
Consensus

RC2G0299600   VHTQSYVSGLEKAMDQASKDGLIFIEGTGPTYATVETFQESLIAAGAGIALIDSVVAESKNSRDPPTGFALIRPPGHHAI   240
RC6G0022900   ................................................................................     0
RC1G0265100   ................................................................................     0
RC6G0533000   ................................................................................     0
Consensus

RC2G0299600   PKGPMGFCVFGNIAIAARYAQRAHGLKRVFIIDFDVHHGNGTNDAFYEDPDIFFLSTHQDGSYPGTGKFDEIGRGDGEGT   320
RC6G0022900   ....MGFCVFGNIAIAAHYAQRAHGLKQVFIIDFDVHHGNGTNDAFYEDPDIFFLSTHQI....................    56
RC1G0265100   ....MGFCVFGNIAIAAHYAQRAYGLKGVFIIDFDVHHGNGTNDAFYQDPDIFFLSTHQVT......IFDTLSVQIYLYV    70
RC6G0533000   ....MGFCVFGNIAIAAHYAQCAHGLKGVFIIDFDVHHGNGTNDAFYEDLDIFFLSTHQI....................    56
Consensus         mgfcvfgniaiaa yaq a glk vfiidfdvhhgngtndafy d difflsthq

RC2G0299600   TLNLPLPGGSGDTAMRTVFDDVIVPCAQRFKPDIILVSAGYDAHALDPLASLQFTTGTYYMLASSIQQLAKDICGGRCVF   400
RC6G0022900   ................................................................................    56
RC1G0265100   LVMLV...........................................................................    75
RC6G0533000   ................................................................................    56
Consensus

RC2G0299600   FLEGGYNLDSLSYSVADSFRAFLKEPSLASTFDNPAMLYEEPSMKIKQAIQRVKHLHS                         458
RC6G0022900   ..........................................................                          56
RC1G0265100   ..........................................................                          75
RC6G0533000   ..........................................................                          56
Consensus
```

Figure S4 Sequences alignment of the four RcHDA14 homologous proteins in rose.

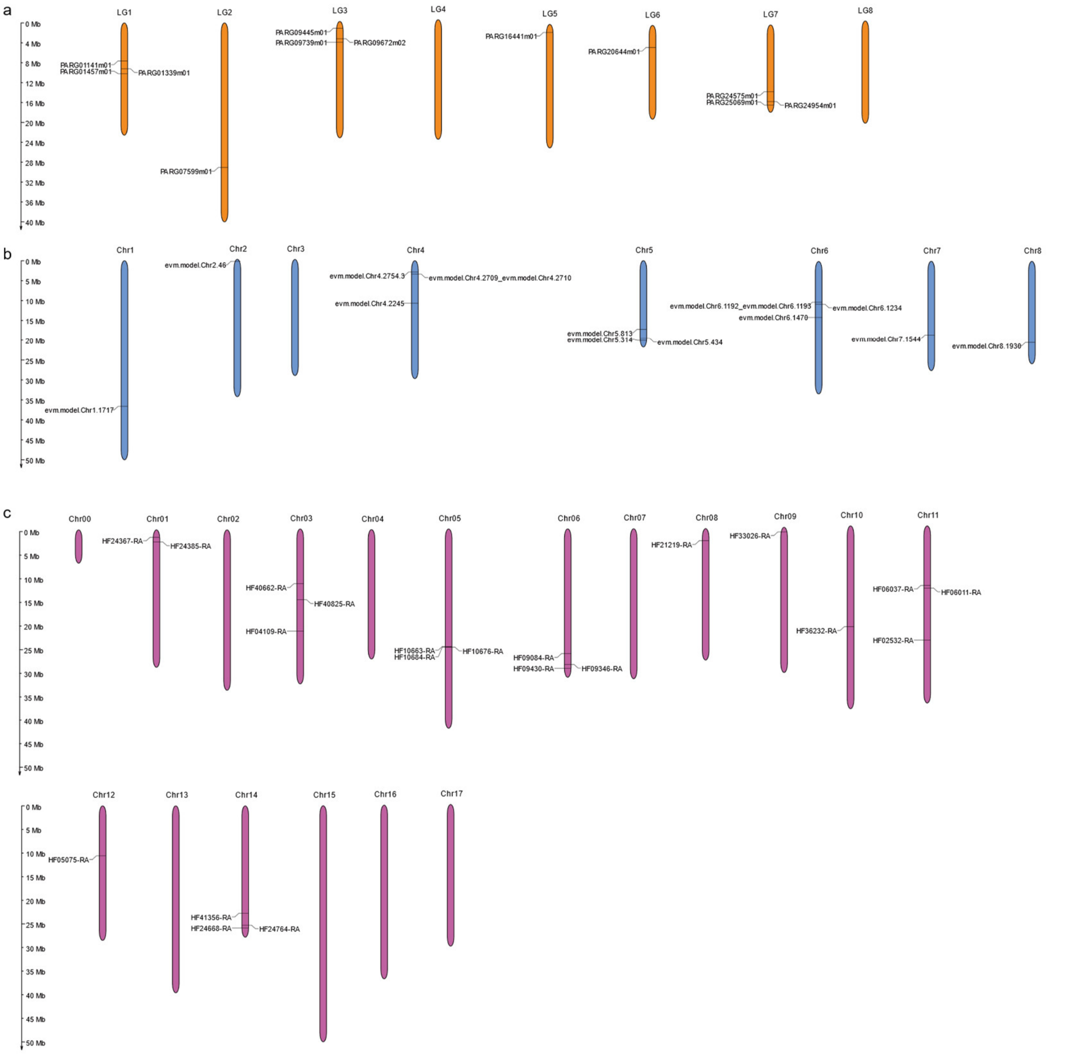

Figure S6 Gene locations of *HDACs* on the chromosome. a.The *ParHDACs* genes of *P.armeniaca* b.The *PyHDACs* genes of *P.yedoensis* c.The *MdHDACs* genes of *M.domenstica*

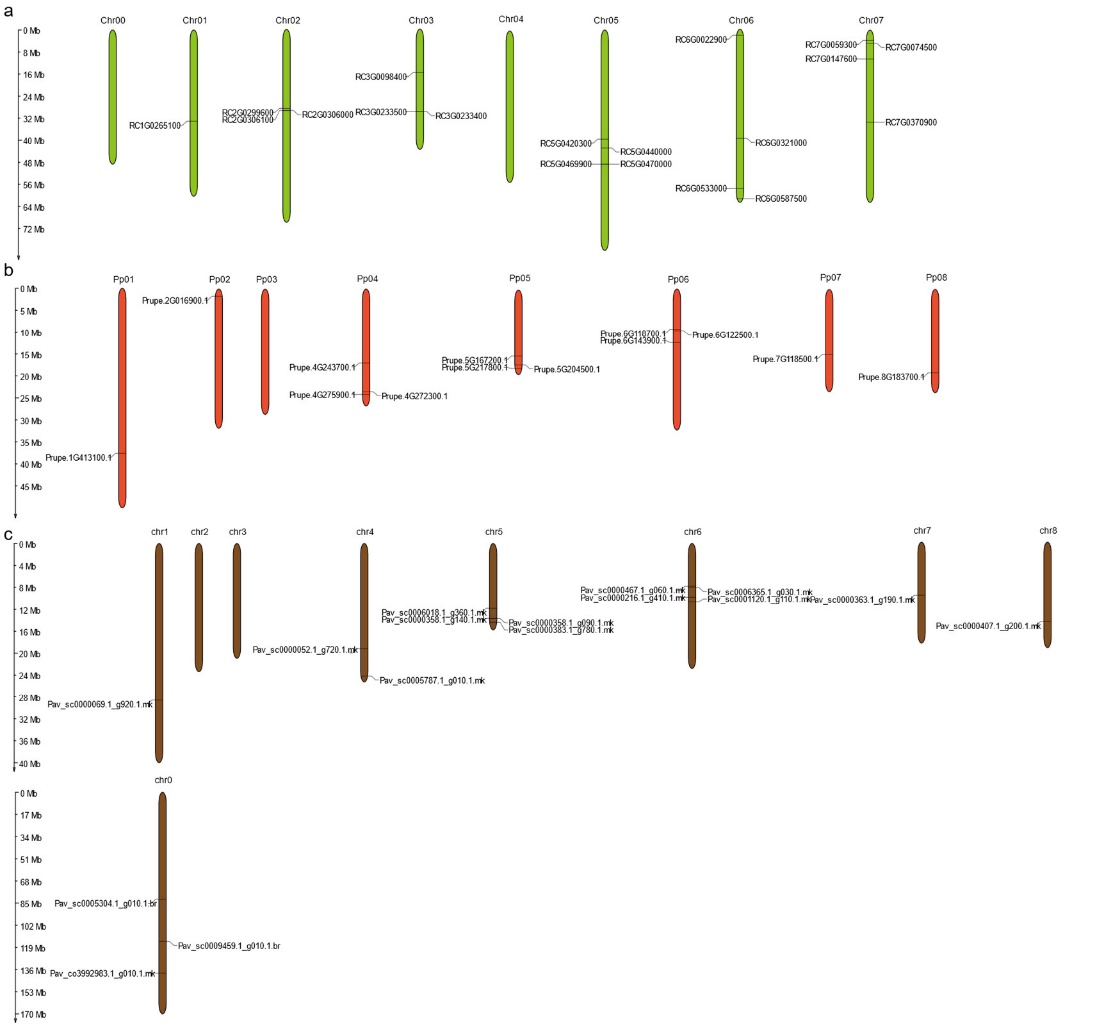

Figure S7 Gene locations of *HDACs* on the chromosome. a.The *RcHDACs* genes of *R. chinensis* b.The *PpHDACs* genes of *P.persica* c.The *PavHDACs* genes of *P.avium.*

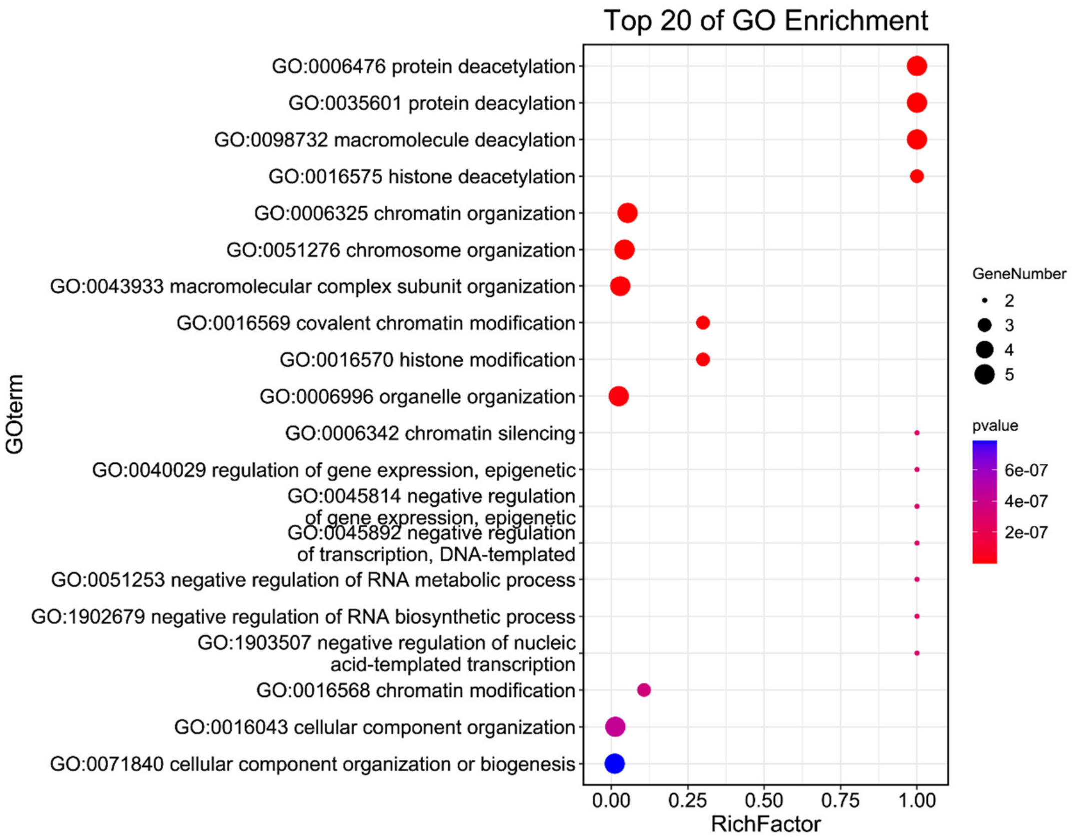

Figure S8 Dot plot of PmHDACs GO enrichment analysis.

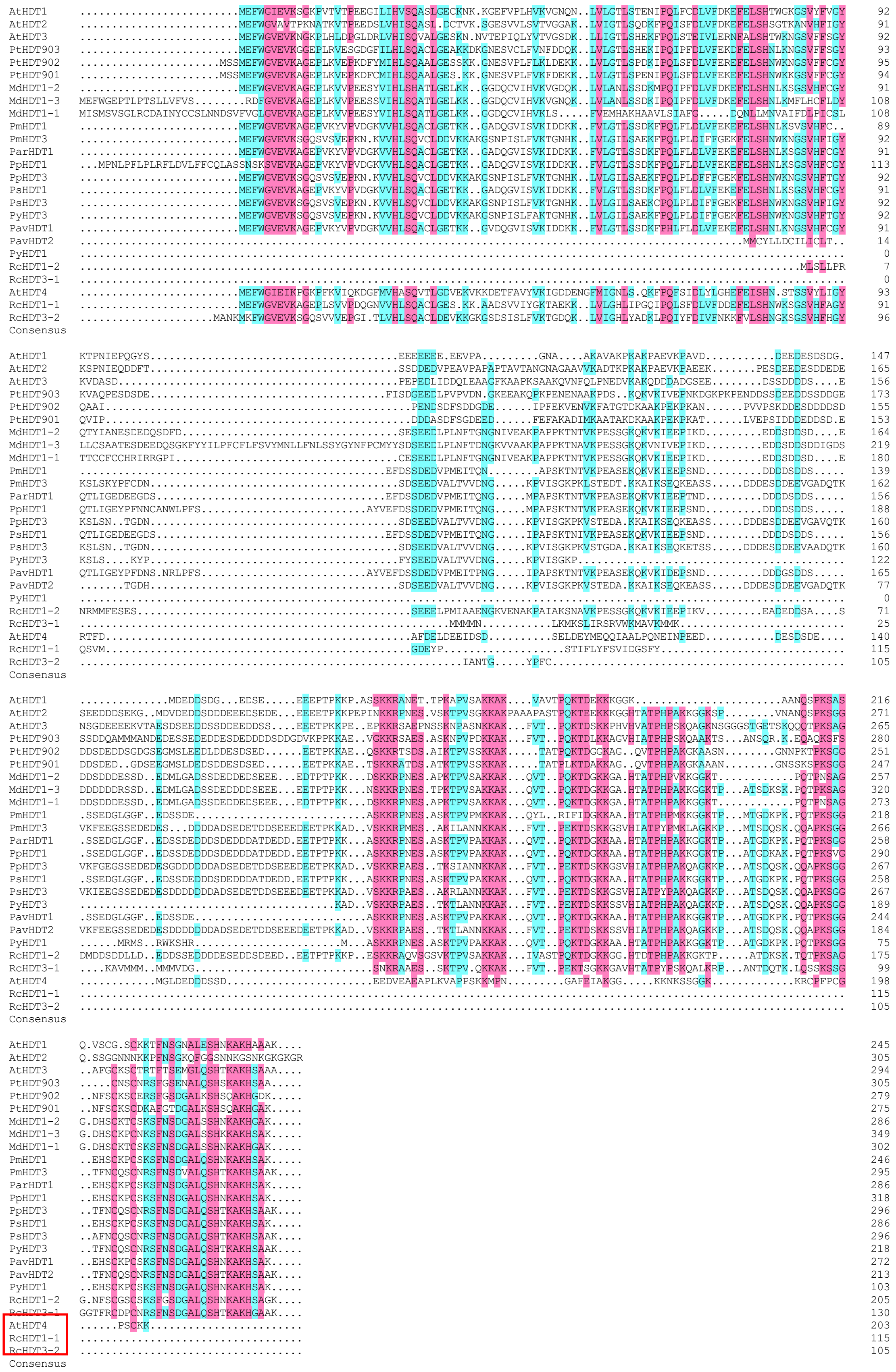

Figure S9 Sequences alignment of all HD2 family proteins in *Arabidopsis*, *P. trichocarpa* and 8

Rosaceae plants.

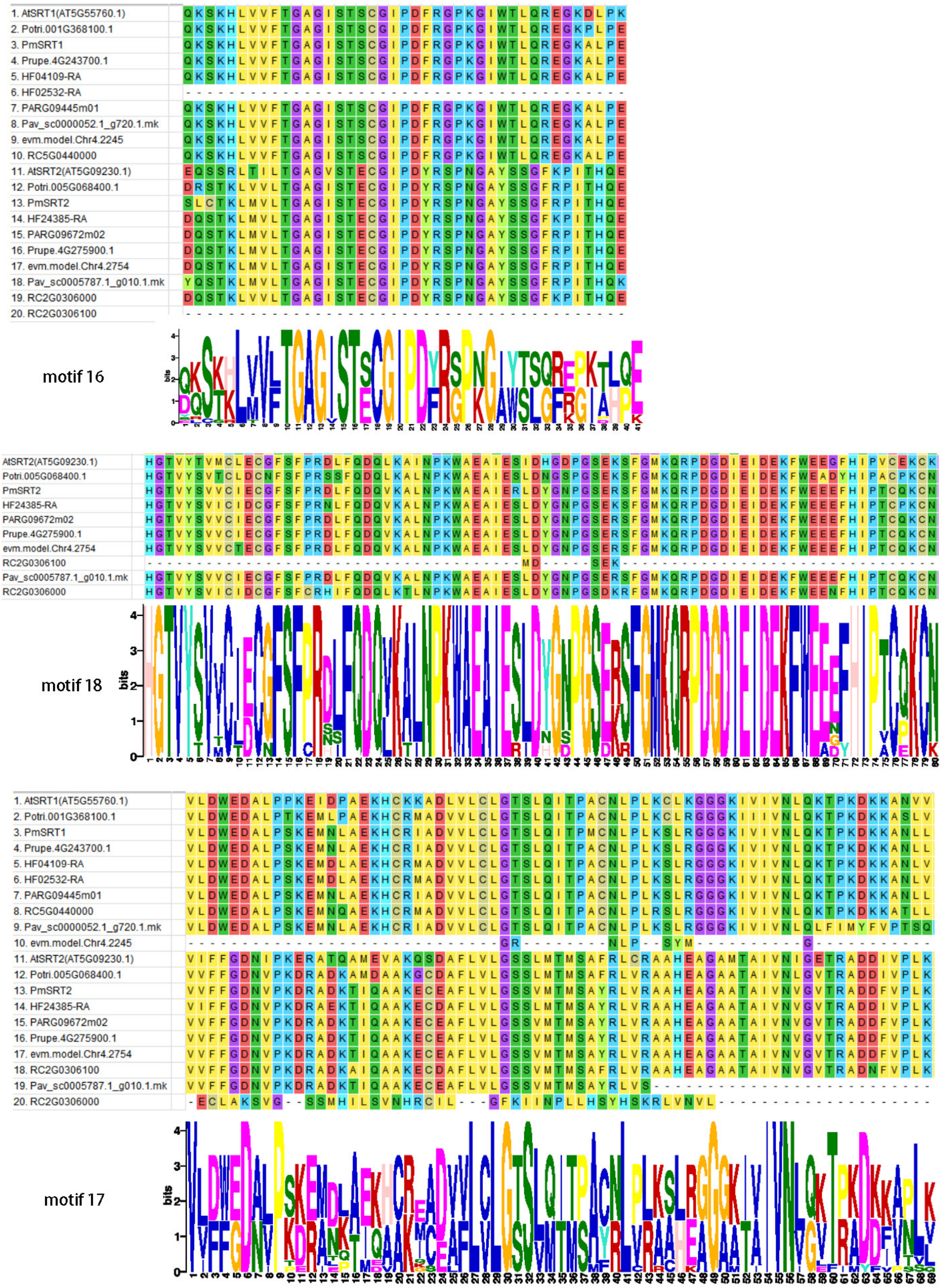

Figure S10 Motif and sequences analysis of *SIR2* genes in 10 plants.
