# Supplementary figures and images for: HDACs Gene Family Analysis of Eight Rosaceae Genomes Reveals the Genomic Marker of Cold Stress in Prunus mume
Source: Int J Mol Sci. 2022 May 25;23(11):5957. doi: 10.3390/ijms23115957 (PMC9180812; doi:10.3390/ijms23115957)

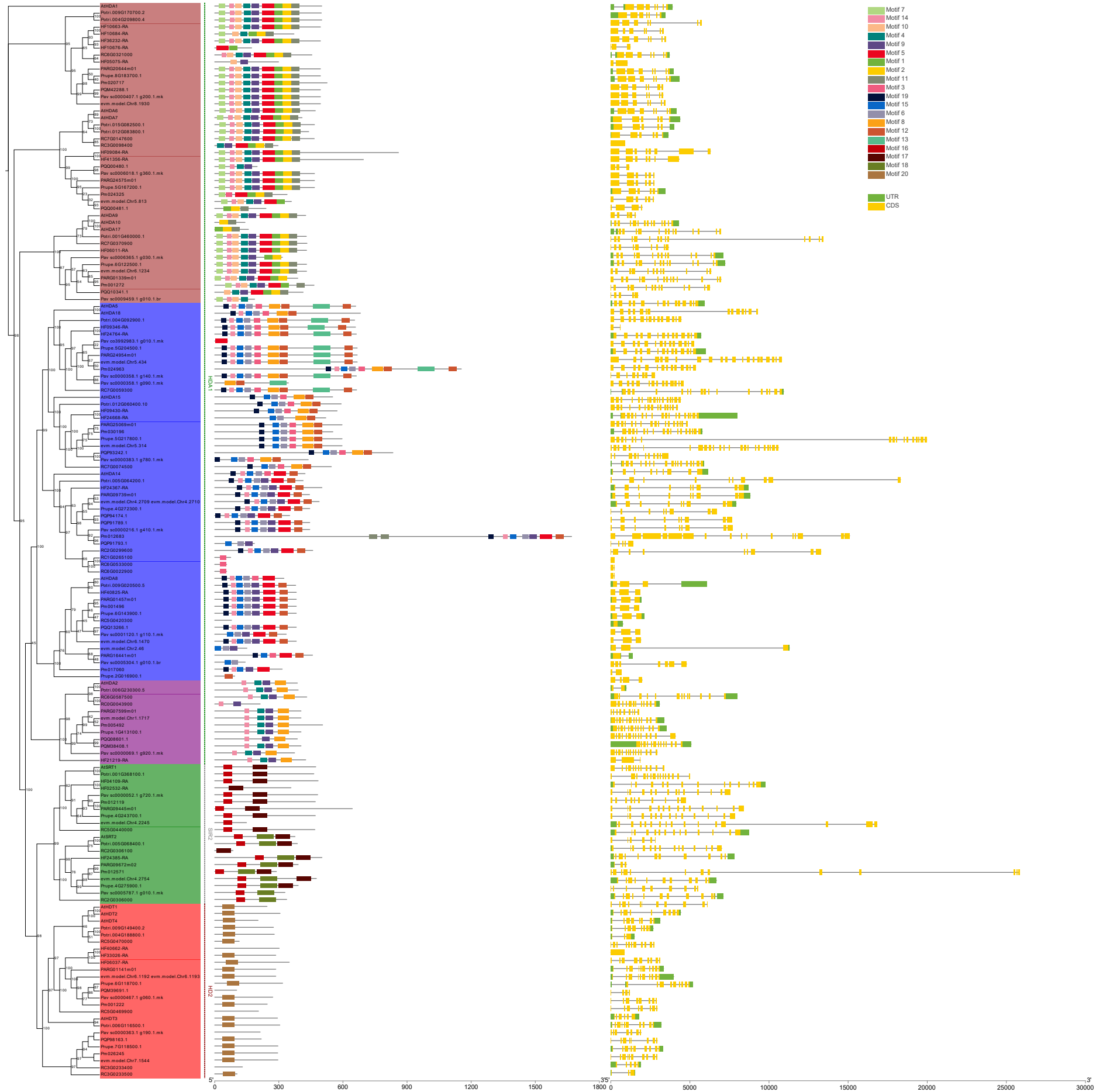

Supplement: Supplementary file 1 [file ijms-23-05957-s001.zip › ijms-1723682-supplementary/Supplementary Materials/Figure S5.pdf]
